# Intensive Care Units Healthcare Professionals’ Experiences and Negotiations at the Beginning of the COVID-19 Pandemic in Germany: A Grounded Theory Study

**DOI:** 10.1177/00469580221081059

**Published:** 2022-05-06

**Authors:** Madlen Hörold, Karl Philipp Drewitz, Julia Piel, Ilona Hrudey, Magdalena Rohr, Vreni Brunnthaler, Claudia Hasenpusch, Angela Ulrich, Niklas Otto, Susanne Brandstetter, Christian Apfelbacher

**Affiliations:** 1 61074 Otto von Guericke University Magdeburg, Faculty of Medicine, Institute of Social Medicine and Health Systems Research, Magdeburg, Saxony-Anhalt, Germany; 2 210419 University of Regensburg, Faculty of Medicine, Medical Sociology, Regensburg, Bavaria, Germany; 3 39067 University Hospital Magdeburg, Magdeburg, Saxony-Anhalt, Germany; 4 University of Regensburg, 210419 University Children’s Hospital Regensburg (KUNO-Clinics), Regensburg, Bavaria, Germany

**Keywords:** qualitative research, frontline workers, COVID-19, intensive care unit, acute critical care, pandemic experience, ambivalence

## Abstract

Faced with the pandemic of the novel coronavirus (SARS-CoV-2), healthcare professionals (HCPs) in intensive care units (ICU) adjusted their organizational, operational, and personal procedures to ensure care for COVID-19 patients. We used grounded theory approach to explore ICU HCPs' perspectives on professional action at the beginning of the COVID-19 pandemic in Germany from March to July 2020. The study aimed to examine implicit principles on negotiating social practice and interaction of ICU HCPs in an exceptional situation, which was characterized by a high level of changes. We conducted theme-guided qualitative telephone/virtual interviews with 39 ICU HCPs from ten German federal states. The data collection followed the principles of theoretical sampling. We adpoted grounded theory approach proposed by Charmaz and discussed using Lüscher’s theoretical concept of ambivalence. The analysis revealed five interconnected categories about the ICU HCPs’ negotiation of social practice and interaction at the beginning of the COVID-19 pandemic in Germany. In this context, a complex field of ambivalence (key category) emerged between habits and routines of a pre-pandemic normality. Pragmatic restructuring processes were initiated, which quickly resulted in a new normality of a “daily routine of preparation”. Dealing with ambivalence offers the potential for change.

**What do we already know about this topic?**

The preparedness of healthcare professionals (HCPs) for challenges like infectious disease outbreaks (e.g., COVID-19) are decisive in determining whether comprehensive care can be provided for critically ill patients.

**How does your research contribute to the field?**

We examined how HCPs constructed the frame of professional action during the preparation and coping phase for the care of COVID-19 patients in Germany, focused on investigating HCPs’ specific experiences, and associated actions.

**What are your research’s implications towards theory, practice, or policy?**

We highlighted a complex field of ambivalence by ICU HCPs between habits and routines of a pre-pandemic normality and pragmatic restructuring concepts at the beginning of the pandemic. We emphasized that ICU HCPs should be/become aware of the dynamics and complexities of the system and seek or engage necessary measures for themselves in managing the new normality of a “daily routine of preparation.”

## Introduction

Since the outbreak of the Coronavirus Disease 2019 (COVID-19) pandemic, health care systems across the world have been facing unprecedented challenges in continuously re-organizing (intensive) care. In the beginning, strategies for preparing for rapidly changing situations of care were accompanied by substantial uncertainty.^1-3^ The German health-care system has first been confronted with the novel severe acute respiratory syndrome coronavirus type 2 (SARS-CoV-2) causing COVID-19 in January 2020.^4^

The extent to which healthcare professionals (HCPs) are prepared or can be prepared for unforeseeable, dynamic changes and their impact on the care situation are decisive in determining whether comprehensive care can be provided for critically ill COVID-19 patients. By increasing abilities to provide intensive care to patients, hospitals established specialized COVID-19 intensive care units (ICUs) or expanded capacities.^5,6^ HCPs from across the hospital were utilized to staff these ICUs.^6^ As a result, HCPs had to adapt rapidly to new workspaces, colleagues, policies, and treatment protocols. Recent studies have shown that ICU HCPs experienced high levels of psychological and physical burden during the pandemic.^7-9^ Zhang et al. revealed the process of frontline nurses’ psychological changes and showed the pattern of ambivalence, emotional exhaustion, and energy renewal.^10^ Sociology understands ambivalence as a temporary or permanent irresolvable situation, which leads to contradictions in feeling, thinking, and acting or in the social structures of the involved individuals due to competing action-guiding values and their evaluation. Ambivalence can be a basic concept in the reconstruction of social practice.^11,12^ It is thus of essential importance to identify the strategies that HCPs on ICUs are developing within their institutional environment to maintain the quality of professional care, to find out what decisions are made and which processes are initiated to re-adjust workflows and to provide appropriate care to patients under the conditions of the pandemic. To address this research gap, we used grounded theory to explore ICU HCPs’ perspectives on professional action at the beginning of the COVID-19 pandemic in Germany from March to July 2020. The main research interest of our study was to examine implicit principles that negotiated social practice and interaction of ICU HCPs in an exceptional situation, which was characterized by a high level of change.

## Material and Methods

### Methodology

At the beginning of the pandemic outbreak, our research team was approached by ICU clinicians with the idea for this study. The research team developed the study protocol collaboratively. We explored how HCPs brought their professional actions into a meaningful order under the circumstances of the beginning of the COVID-19 pandemic in German ICUs. We used the grounded theory approach proposed by Charmaz^13^ to develop an interpretative analysis of the data material. Grounded theory studies focus on social processes or (inter-)actions: The constructivist grounded theory (CGT) emphasizes the shared meaning constructed by both the participant(s) and the researcher(s). In doing so, studies focus on what happens and how people interact in relation to the phenomenon under research. To make links between categories visible (axial coding), we used a coding frame and asked questions to the material include (1) conditions, the circumstances or situations that form the structure of the studied phenomena; (2) actions/interactions, participants' routines or problems; and (3) consequences, outcomes of actions/interactions.^13^ The CGT in our study led us to understand the implicit principles of negotiating of social practices among ICU HCPs in the phase of the incoming COVID-19 pandemic. In accordance with grounded theory approaches, we integrally related sampling, data collection and data analysis.^13,14^

### Recruitment and Participants

We contacted 129 hospitals or individual HCPs throughout Germany (across all 16 federal states) by different channels (e-mail, telephone, professional networks, distribution of a flyer) and personal contacts via snowball sampling^15^ between end of March and mid July 2020 for a qualitative interview. The idea-driven ICU clinicians supported us during the recruitment process (snowballing). Patients or the public were not involved.

We addressed persons from several healthcare professions, for example, physicians, (academically qualified) nursing staff and medical students in German hospitals, who were involved in the clinical acute care of COVID-19 patients requiring intensive care or monitoring. All participants were informed in advance about our publication strategy, which is in line with our study protocol. Two contacted individuals actively declined to participate in the study at the first point of contact without specific reasons, while two asked to be contacted only after the pandemic, 39 agreed to participate. All other contacts did not respond or did not get back in touch after initial communication. There was no drop-out of participants between recruitment and the actual interview. To gather rich data^13^, we aimed for a heterogeneous sample both in terms of individual characteristics (e.g., work experience, gender, social and ethnic origin, educational background) and the professional environment (including the level of care provided by the hospital). Through comprehensive data analysis, we aimed at providing insights on changes in the pandemic experience of ICU HCPs and related negotiation processes. This article focuses on how ICU staff reorganized and initiated new normalities in social processes during the beginning of the pandemic.

### Data Collection

Under the circumstances of the pandemic, the most appropriate approach to assess HCPs’ negotiation and interaction processes at the beginning of the first wave was to engage low-threshold offers for conversations. It was not possible to talk to them in the field, so we chose theme-guided qualitative telephone/virtual interviews structured as openly as possible. Therefore, we developed a thematic interview guide (see supplementary information) based on the most relevant emerging issues and discussion points of the federal government, federal states governments, global research activities and public opinion regarding the challenges of the pandemic. This semi-structured approach supported the different interviewers in keeping the focus on the research interest and, if necessary, in responding adequately to specific events in the interview situation. Participants were initially asked about their personal perspectives on professional action at the first wave of the COVID-19 pandemic. The open beginning of the interview gave the participants the opportunity to create own narratives on the topic.^16^ They set their own relevance, to express the subjective meaning of the topic and to reflect on what they have experienced. Seven researchers conducted the interviews. All interviewers were female with a varying degree of experience in qualitative research. Four had prior experience in healthcare (one nurse, one radiographer, one psychologist, one physiotherapist). We saw great potential in the heterogeneity of the research team for grounded theory practice. For example, more experienced researchers briefed the others to conduct interviews. By involving multiple researchers in the process, we ensured the trustworthiness of the findings and controlled biases.^17^ The interview guide was tested in two interviews. No modifications were made. When inviting participants to take part in the interview, we asked them to share an artifact (newspaper clipping, photo, picture, etc.) that represented their personal experience on the pandemic. Only one participant sent us a photographic self-portrait after the interview, which showed her with personal protective equipment at her workplace (ICU).

In order to contextualize the insights gained from the interviews, socio-demographic data, information on professional biography, the current situation within the current participants' workplace and various aspects of the interview situation, like atmosphere and interaction, were collected as data of secondary order.

### Data Processing, Analysis and Reporting

In our approach, we followed the standards of qualitative research.^18^ The data collection was based on the principles of theoretical sampling. Charmaz describes theoretical sampling as a process of “starting with data, constructing tentative ideas about the data, and then examining these ideas through further empirical inquiry.”^13^ We continued the sampling until no new codes emerged to saturate our categories.^13^ The coding and interpretation team consisted of the interviewers and three additional researchers, who had prior experience in conducting qualitative interview studies.

The quality and methodically controlled procedure of inductive data analysis was achieved by communicative validation within interpretative group sessions through regular meetings via a video conferencing system.^19^ In our sessions, we used software tools (either ATLAS.ti or MAXQDA, due to different institutional availabilities) for data management and coding.^13^ The use of a virtual team workspace (the wiki software “Confluence” by Atlassian) enabled us to effectively store and structure memoing and coding in a tabular form, to share insights, and to develope visualizations regardless of the researchers’ location.

We reflected on interactions during the interviews and interpretation of the data. We switched between line-by-line, in vivo, incident-to-incident (initial coding) and focused coding on the data.^13^ Memoing on the interpretation of the data was conducted together during the interpretative group sessions. This helped us to develop conjectures and transferred into codes and categories.

Using software tools simply supported the virtual interpretative process with the CGT. Data collection and analysis were conducted in German. The referred quotes in the “Findings” section were translated into English for this article.

### Ethical Considerations, Data Protection and Privacy

We received ethical approval for our research from the institutional review boards of the University of Magdeburg (51/20) as well as the University of Regensburg (20-1771-101) before we performed the first interview. All study activities were conducted in accordance with the declaration of Helsinki^20^ and in compliance with the relevant legal regulations. Each participant gave written informed consent before we made an appointment for the respective interview. We interviewed participants individually via telephone or an appropriate video conferencing system and recorded the interview in an audio format compliant with the General Data Protection Regulation, GDPR.^21^ We assured confidentiality by pseudonyms (five-digit number and fictional name) for each participant. To further fulfill GDPR compliance, we set up a Trusted Third Party to store written informed consent and personal data separately from the research data, as well as to process contact information for requesting a second interview.

## Findings

### Study Sample

The sample consisted of 39 HCPs: 19 nurses, 17 clinicians and three medical students (with prior professional training as a nurse) from ten German federal states. Almost half of the participants were female (n=18). All participants were involved in the acute care of COVID-19 patients in hospitals and had a mean professional experience of 15 years. We conducted the interviews between April 6^th^ and July 13^th^ 2020. They lasted from 12–66 minutes. Further details on the characteristics of the participants are shown in Table 1.

**Table 1.** Characteristics of the Study Participants.

	pseudonym	age	working experience (years)	position and care level acc. to Bormann and Swart^22^	characteristics (in vivo)
1	Peter Distelmeyer (m)	30	3,5	ICU clinician in training, basic and regular care hospital	“[I made] very encouraging and satisfying experiences.”
2	Elisabeth Huber (f)	42	15	ICU senior clinician, centralized care hospital	“You’re making decisions for the safety of the whole team.”
3	Christoph Faber (m)	43	17	ICU clinician, regular care hospital	“[…] it was a time of many yeses, when everybody said: Sure, we’ll do it that way! [A time] when there was less discussion.”
4	Helene Wustrow (f)	38	10	ICU clinician, basic care hospital	“And for my nurses in the beginning, it was like, ‘Yeah, and if you get infected, you’ll die,’ and that’s kind of-. The thought had to get out of their heads.”
5	Jens Mantel (m)	44	17	ICU senior clinician, maximum care hospital	“So it was (.) very hectic during that time. Um, because new structures had to be created constantly and new problems came up constantly. And then there was this outbreak situation […] in the clinic there was […] restlessness […].”
6	Kristin Baumann (f)	26	3	ICU nurse, maximum care hospital	“Because I’m now working on this Corona ward, I know that this is quite good, the staff were asked whether it would be okay for them to work with infected patients.”
7	Uwe Michaelis (m)	53	25	ICU senior clinician, head of the internal ICU, maximum care hospital	“[I] almost fear that [health care professionals] will be forgotten again in three or four months for all the work that has been done.”
8	Bianka Beyer (f)	23	2	ICU nurse, maximum care hospital	“Sometimes you just had the feeling that everybody who doesn’t have corona falls (…) a little bit behind […]”
9	Karsten Steffen (m)	52	26	ICU senior clinician and deputy leader emergency room, centralized care hospital	“We were not initially able to protect the staff in the way that was necessary.”
10	Karl Mohn (m)	59	34	ICU senior clinician, regular care hospital	“It’s a bit like being stranded by plane in the desert somewhere.”
11	Herbert Meister (m)	57	35	ICU nurse, ward manager, maximum care hospital	“There were real troops that ultimately swept through the ward and turned them over one by one. And that has-. Yes. Honestly, I experienced more of a sense of optimism than depression.”
12	Stefan Meihofer (m)	54	27	ICU head clinician, centralized care hospital	“A colleague of mine […] He bought a 3D printer a year and a half ago and immediately sat down at home and started it up, so that in principle we only had all the other - um, what do I know about colleagues - uploaded something (pause) makeshift masks on YouTube and we already got them [ready]. […]”
13	Thomas Steiner (m)	45	17	ICU head clinician, maximum care hospital	“I would never go to the Congo or anywhere to get any SARS, MERS or EBOLA or hemorrhagic fever, I’m way too scared of and [I am] also not tired of living, but that’s something I can avoid, I can go volunteer for that. Here [in this situation], I can’t volunteer.”
14	Malte Efferz (m)	34	>6	Ward manager, centralized care hospital	“There has been excellent collaboration on all of these restructuring and organizational issues.”
15	Samuel Geib (m)	28	4	ICU nurse and medical student, maximum care hospital	“We had a few things left over from the Ebola situation. And we put on a hazmat suit, so to speak. And two to three layers of gloves accordingly. A face visor. Um, so as not to have any surface exposed. It was also one of those moments where I really took a shower for the first time during a shift […].”
16	Felix Lanthaler (m)	38	13	ICU clinician, maximum care hospital	“I think we’ve used all opportunities we’ve had here in the house [clinic] to prepare for this.”
17	Tina Hirsch (f)	34	14	ICU nurse, Helicopter Emergency Medical Services Technical Crew (HEMS-TC), maximum care hospital	“I don’t know what they [nurses] are always afraid of, that’s a problem, because you can get a job in nursing anywhere today, but we are so submissive and the lobby of the physicians is so big. Um, but if we don’t use the opportunity now, then we don’t need to use it anymore, because then we’ve wasted it. So we have to make an effort now.”
18	Heidi Schneider (f)	25-30	5	ICU nurse, non-medical student, maximum care hospital	“Currently it works, but the hygiene guidelines are also adjusted so that the [equipment] also has to be enough. That means I take one mask, throughout the day, for all patients. Which is certainly not standard, under normal circumstances [not] hygienic, and above all most were afraid or also themselves that they themselves are not protected enough, because we would normally take one mask for one patient round […] and currently as I said for eight hours. At the beginning it was very, very uncertain ‘am I still protected at all if I only wear one mask for so long?’ Beside the fact that germs or viruses, bacteria, whatever, are transferred from patient to patient, but I think that the team is doing a good job. And the situation is also stable.”
19	Julian Meyer (m)	27	11	ICU nurse and instructor, basic and regular care hospital	“[…] then I quite consciously avoided a bit of contact, did not wanted the contact with other people now and, of course, the whole-, ‘oh God, do I have a scratchy throat now? Do I have temperature?’ You slept badly […]”
20	Anke Bäcker (f)	37	17	ICU nurse, maximum care hospital	“[…] for many [colleagues] this is impossible, they said they would no longer work if there were no FFP-3 masks to put on again […]”
21	Martin Kurtz (m)	42	15	ICU head clinician, centralized care hospital	“[…] We are prepared […] there I am glad that we know in the future, we simply have a plan that has really proven itself in the crisis, it works.”
22	Rebecca Amann (f)	28	1	Clinician in training in internal medicine, centralized care hospital	“[…] when you have a car crash and you’re like in the last seconds and you want to brake and the wall is getting closer and closer and you realize the brakes aren’t working […]”
23	Helena Kluge (f)	20-25	6	ICU nurse and medical student, maximum care hospital	“So I have demands on myself and if I can’t keep them, because of course everything is an unfamiliar situation for me, then it’s difficult for me when I have to do things that I learned differently.”
24	Kerstin Jäger (m)	42	24	ICU nurse, centralized care hospital	“This is my third world war.” “The weakest link in the chain is the little nurse.”
25	Stefan Bieber (m)	28	9	ICU nurse and ward manager, regular care hospital	“[…] but regarding the implementation, uh, the plan (.) is good but the implementation is miserable. So and that’s why there were already [COVID-19] cases now […]”
26	Gerald Schröter (m)	58	31	ICU head clinician, centralized care hospital	“[…] You stand, because if you are somehow the head of a ward, you are, to a certain extent, autonomous. That means, of course, you have to justify yourself in your immediate environment, but you are still-, you can go through your therapy with a certain self-confidence, I would say. That’s true, of course, that during the COVID time-, there was always a certain insecurity, so one felt observed, because it was also a ward that was composed of several-, that was composed of two clinics. […]”
27	Margret Müller (f)	55	34	ICU nurse and ward manager, centralized care hospital	“That was nevertheless exciting to see how now this coronavirus, I’ll say, amplifies a completely different kind of anxiety all over again.”
28	Svenja Fischer (f)	40	13	ICU nurse, centralized care hospital	“The first bad thing for us was actually that we had to take austerity measures during this time.”
29	Hanna Läufert (f)	50	23	ICU nurse and deputy ward manager, centralized care hospital	“[…] then you first went home [after work] and didn’t turn anything on at first. So I didn’t do anything. I didn’t turn on the TV, I didn’t turn on the radio, I said, ‘I can’t listen to it anymore.’ Because it’s just so out of reach.”
30	Carolin Kunze (f)	35	>10	ICU nurse, maximum care hospital	“[…] I hope and really pray all the time that it won’t be as bad as in Italy or France. That is actually my big hope at the moment. That I think it’s not going to be that bad. We are in Germany - we are, we have a reasonably good health care system.”
31	Allessandro Ducanto (m)	45‐50	14	ICU senior clinician, centralized care hospital	“[…] We worked like this before in the team, we work with anesthesia nurses and one of our fears was the risk for the nurses. So we can protect ourselves but it is also clear to protect the nurse, too. Right? And the new protocol for intubation for example was developed together with the nurses.”
32	Sebastian Hansen (m)	32	>5	Clinician internal medicine, basic care hospital	“We are developing a hygiene concept, we are developing a concept on how to deal with these kind of patients. And that took a lot of uncertainty out of it. And this structure took also, at least for me, away my fear.”
33	Sophie Schünemann (f)	49	26	ICU nurse and ward manager, maximum care hospital	“[…] there are certainly doctors who of course always said ‘Of course we will also go into this room protected and care for the patient’. But then there are also the doctors who sometimes said um ‘I’ll wait until the result of the test is there'.”
34	Mark Schröder (m)	23	3	ICU nurse, basic and regular care hospital	“And the donning and doffing every time before that, that was just always, [until you] got the certain handling out of it, how best to don, how best to doff then-, well, how best to doff hygienically. Then the hands. So, that was already a change.”
35	Christian Trüb (m)	30	14	ICU nurse, HEMS-TC, maximum care hospital	“So, because in the circle of my acquaintances, or friends, almost no one adhered to this contact ban. And that’s what I did. And when it was mentioned, there tends to be discussion. And, yes, I think everyone who doesn’t work in the medical field, who doesn’t work in nursing and doesn’t have anything to do with it directly, doesn’t take it seriously, at least that’s my feeling. Because it’s just a flu virus, like that […]”
36	Markus Feger (m)	33	7	ICU clinician, maximum care hospital	“[…] we are still waiting for the wave, so to speak. We hope, of course, that it won’t come.”
37	Doris Landau (f)	27	8	ICU nurse, maximum care hospital	“And now, of course, they [clinic management] want you to step up and leave everything on the side and just come rumbling into the clinic. And from that, I’m like: no, honestly.”
38	Silvia Sorge (f)	49	30	ICU nurse, maximum care hospital	“That means, of course, if we have in the back of our minds, if the COVID-19 situation escalates, we will actually be at the end, yes. That’s what also depressed us a bit in this situation […]”
39	Melanie Munz (f)	30	8	Nurse and medical student, maximum care hospital	“[…] I started to work, I just started to sweat super fast, that’s just very uncomfortable to work in these clothes and um also very strenuous. I meanwhile drink up to four, five liters of water on the ward, um change my clothes three times per shift, depending on how strenuous it was and how much we had to do, um but just um one hour in these isolation clothes is enough that you are sweating down to (laughs) your underpants. […] so this, this kind of working, this, this silence [in the patient room] and that you are actually not really tied to the outside […]”

### Managing Ambivalence and Negotiating Social Practice and Interaction

**Figure 1.**
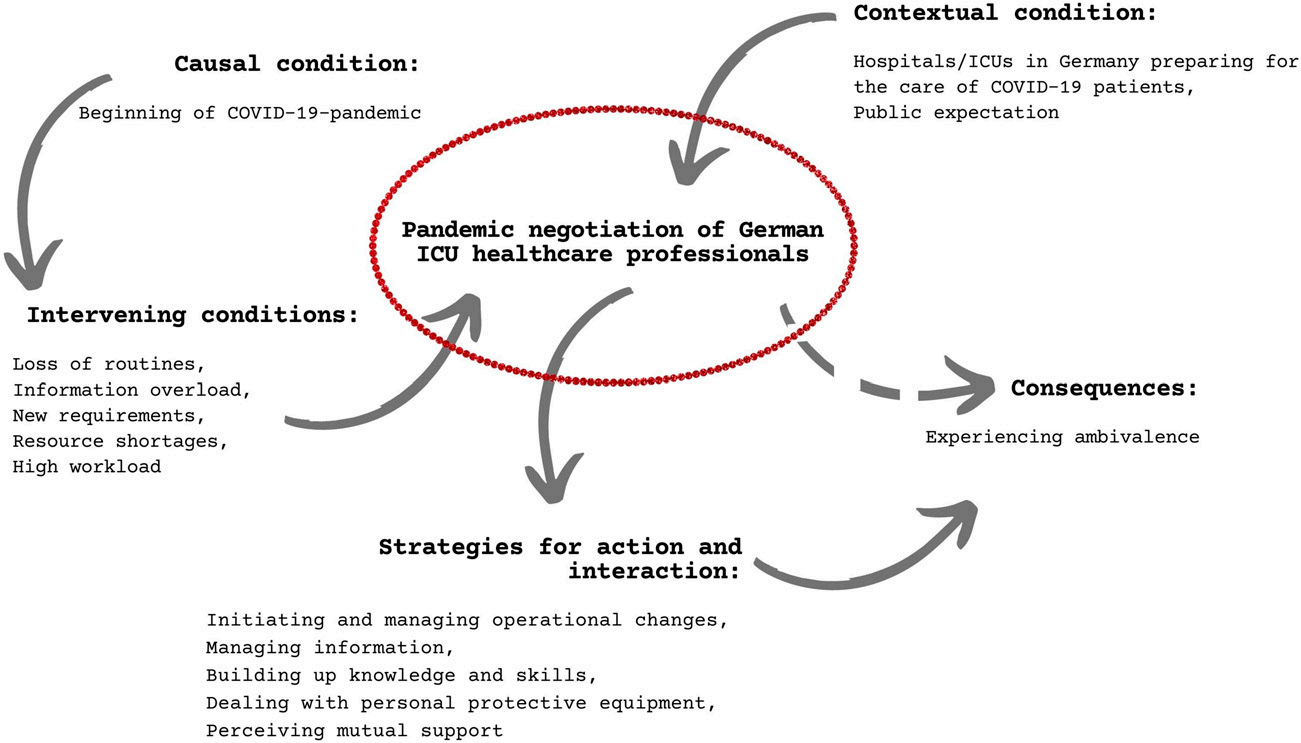
Processes around ambivalence and its embedding in the social practice and interaction of ICU HCPs at the beginning of COVID-19.

In our data analysis, we focused on the negotiation of professional action as the central social practice of ICU HCPs (Figure 1). In this preparation phase, we observed a complex field of ambivalence raising conditions that challenged, but also maintained and/or reinforced negotiation of professional action. Ambivalence among ICU HCPs ensured that (inter)actions were constantly renegotiated. Realizing that most hospitals were not prepared for a pandemic, ICU HCPs nonetheless demonstrated accountability for the situation and to hospitals. The emergence of a novel disease (COVID-19) revealed the experience of ambivalence. Thus, ICU HCPs perceived public expectations to be professional medical authorities and to provide high quality critical care.

Staff from other wards supported ICU care. HCPs in the ICU care participated in the preparation processes and assumed responsibility, for example, in task forces. They were supported in negotiating social practices and interactions by ICU and hospital management.

The iterative analysis of the processes around ambivalence and its embedding in the social practice and interaction of ICU HCPs at the beginning of the COVID-19 pandemic were described afterwards.

The codes were stored into five categories mapping different social processes: initiating and managing operational changes, managing information, building up knowledge and skills, dealing with personal protective equipment, and perceiving mutual support. Ambivalence was a recurrent subject within each of these categories. In the following sections, we present ambivalence within these five process domains.

### Ambivalence in Operational Changes

The category “Initiating and managing operational changes” specifies processes for implementing COVID-units and new workflows, as well as managing human resources. In the hospitals, multidisciplinary crisis teams or task forces were set up to extend the hospital management, which centrally determined the measures for their facilities (based on legal requirements).

“We found out that our number of intensive care beds won’t be enough, […], also the anaesthesia equipment won’t be enough, so in the end I [tried] to organize equipment from the homecare area that is licensed for invasive ventilation therapy [in the hospital].” Elisabeth Huber, senior clinician

This excerpt from the interview with a clinician exemplifies that the crisis teams autonomously supplemented institutional measures with daily strategies to optimize care processes. Using “we” perspective (“we found out that”) indicates that at the sub-institutional level, the teams found own solutions in collaborative processes of reflection and action to compensate institutional deficiencies. Their decisions had a strong impact on structures and operational processes.

Furthermore, participants reported on the organizational measures taken to increase capacities and to reorganize workflows in their units through operational changes. The loss of routines triggered uncertainties.

“It was more problematic in this chaos (laughing), where people were divided into new intensive care units, where they had to see for themselves, […] there are students as assistants, new stand-by persons, but also people who want to be trained, both medical and nursing, that there suddenly was a huge amount of people on the intensive care units and that it was not easy to manage.”—Elisabeth Huber, senior clinician

Several participants described restructuring processes through images of building and crafting as “raising walls,” “modularizing,” and “assembling” units. For example, one clinician illustrated team restructuring by referring on manufacturing:

“In our case, a […] team consisted of four people, namely a team lead […], which was an intensive care nurse. The second companion was a clinician, who is experienced in intensive care, but I deliberately did not give him the lead; instead, the lead was with the intensive care nurse. Then a clinician who can intubate and do critical care. The third hand was still a nurse, […], but not necessarily […] a specialist nurse intensive care. The fourth hand was a […] medical student in his final year. So we consistently planned these four […] then we modularized that.”—Martin Kurtz, head clinician

In a “chaotic” situation, the participants experienced contradictions: They described a high motivation and commitment of the HCPs to help in the newly established routines of collaborative processes. While hierarchical structures between positions or career levels were relevant regarding the transfer of information and new team-building principles, they became less relevant in teamwork itself.

Thus, ICUs were extended (partly also by structural measures), regular wards were closed, merged, medically rededicated or equipped with other medical technology. Decisions were made pragmatically and quickly in order to organize collaborative, inter-professional action in preparing the unit for an expected high number of COVID-19 patients. For example, one clinician mentioned on the (re-)use of all available equipment:

“We then divided two stations in the house. A monitoring station and a cardiology monitoring station. Then we upgraded them with at least some inferior home ventilators and respirators that were still available.”—Karl Mohn, senior clinician

The clinician presented preparing the expected situation as a jointly experienced process (“we selected”; “we upgraded”). The use of space and material previously considered infeasible, became possible and established during this phase, *“many people around [me] were so solution-oriented”* (Herbert Meister, nurse). Even realizing inconceivable actions became a common experience for HCPs.

In order to increase personnel capacities, HCPs were reallocated, qualified or recruited (with and without previous medical/nursing experience).

The head clinicians and leading nurses showed a high responsibility for their staff. They were concerned to protect them, as specified in the following quote:

“[…] and then I get this mechanism, don’t make it worse. So it means, um, protect your team, protect that no one gets infected.”—Thomas Steiner, head clinician

In this example, responsibility was expressed by implicit behavior (“mechanism”) and retrospectively reflected by an inner voice (“protect your team”). The protection of the team determined the clinician’s actions on his ward.

Besides this one, other cases also showed that leading staff acted as regulating authorities on the wards, although they faced various uncertainties about the virus at the beginning of the pandemic. Some experienced their staff as insecure and partially defensive about working in the immediate care of COVID-19 patients.

“[…] we drew lots together to decide who would now be responsible for the COVID patients and, um, these were highly dramatic scenes, so that our nursing management actually had to delegate staff because they were not in a position to organize themselves, for example, to sort things out.”—Thomas Steiner, head clinician

Drawing lots for COVID-19 patients served as a pragmatic strategy to quickly find a solution regarding staff distribution. This strategy induced great fear of infection among ICU staff at a time when scientific knowledge was scarce and already dynamic.

Besides making decisions about the allocation of HCPs in the care of COVID-19 patients, the ICU management had also to decide on the implementation of further training structures for non-specialized staff. A clinician reported:

“Another important point was the training and recruitment of new employees or even the redistribution of employees from other departments […], which were not familiar with the internal medicine. Surgeons worked with them, neurologists worked with them and the same applied to nursing care. In the end, the staff had to be trained both in the normal ward and in the intensive care unit, as well as the staff who worked in the intensive care unit.”—Elisabeth Huber, senior clinician

ICU management also asked for support of volunteers as chaplains or crisis intervention teams who offered talks or short interventions.

The participants also spoke about supporting by colleagues without ICU skills, auxiliaries and service staff and the associated assignment/allocation of tasks, as well as changes in rostering (floater, digitization).

Changes in operational structures also affected everyday routines. Some participants perceived these modifications as stressful. One nurse remembered the organization of food serving for hospital staff as follows:

“So that’s where we were pushed to our limits. I was in a panic in between. I thought, this is my third world war – in my viewpoint. (…) when you’re going to work and you have to wait in line because you want to take a small apple and a pear [for the shift].”—Kerstin Jäger, nurse

The image created in this passage expresses the experienced contrasts in the daily routine on the workplace in the situation. The nurse addressed, that the breakdown of previous routines in the pandemic slowed down processes, which in turn led to frustration. Besides changed and extended care tasks, daily activities were also complicated by trivial circumstances. Some nurses, for example, mentioned that ward rounds were carried out without direct contact between clinicians and patients. However, we observed ambivalences in the clinicians’ reports, for example about assuming nursing responsibilities.

“Well, to be honest, I have to say that I can’t really do some of it very well. I never learned real personal hygiene, that is, I could only do that as an assistant, if you will. […] for me personally (it has) not been a problem at all. But it is of course finally a waste of medical resources, if you wanted to transfer it now, for example, into the normal working day […].”—Peter Distelmeyer, clinician in training

The clinician presented the assumption of nursing responsibilities as personally manageable, while at the same time looking critically at the use of clinical resources.

### Ambivalence in Information Management

The category “Managing information” consists of three concepts, which integrate related processes: “Being confronted with flood of information with limited validity,” “Exploring new digital world,” and “Talking and listening.” We observed ambivalence in different experiences on the accessibility and receptivity of information.

The participants talked about their efforts in collecting information and difficulties in managing its large amount (via handout, e-mail and telephone). Within a very short time, HCPs in leadership positions prepared work instructions and process manuals and had to revise them.

“Then, of course, it’s really extremely difficult, because you get about fifty e-mails a day. So, of course, you are also flooded with mails, which you really try to read properly. […] then I had once not read a sentence properly, then I immediately got scolded whether I have not read this, […] I think such information you should be much shorter and somehow reduce to the concise […] that was just too much.”—Sophie Schünemann, nurse, ward manager

They filtered out changes that were most relevant to social practice in ICU teams and continued to experience this challenge every day.

Participants spoke about an increasing use of the intranet, about video messages and instructional videos up to the development of apps for rostering. They mainly used digital tools to manage the mass of information. In doing so, they proved to be creative and solution-oriented.

“[…] I made a fool of myself and shot a video for the whole staff, which was on the intranet and then always ran.”—Martin Kurtz, head clinician

Role requirements expanded in some positions. By acting like a “fool,” HCPs in higher positions suddenly showed a personal side.

Some HCPs reported a lack of transparency, as they were not involved in the decision-making and planning processes and were not informed about results. This increased feeling of insecurity, addressed by a nurse:

“In our hospital, a so-called task force was established relatively quickly, in which hygiene, the head clinician and the leading nurses of the various wards concerned got together and tried to work out a concept, which admittedly was not entirely transparent. So there were-, they met every day and discussed every day. But an ordinary employee like me, I would say that it was not very transparent, so that at times I felt a bit insecure, because every day there were innovations, but, yes, without explanation.”—Julian Meyer, nurse

Experiences with the communication of measures by decision-makers to ICU staff varied widely, depending on the institutional communication culture and other factors. Generally, participants felt that communication processes, especially in the preparation phase, were hierarchically structured. As a result, individuals in “ordinary” positions were confronted with information deficits.

In contrast, ICU leaders (both medical and nursing) mentioned the importance of talking and listening to each other to reduce contradictions. Some of the participants recognized that it was constructive to listen to employees’ needs and concerns, answering their questions and explaining current measures in this negotiation process.

“In principle, we tried to talk to people every day and every hour and every minute, so that no one would have the feeling that they were left alone with the problem. But it could not be solved immediately.”—Karsten Steffen, senior clinician

Consequently, the situation was considered as a common problem to be managed together. The processes of talking and listening to each other were therefore characterized by a willingness to act and stay motivated despite the challenges and ambivalent experiences.

### Ambivalence in Learning New Skills and Using Old Equipment

The category “Building up knowledge and skills” consisted of the three process-related concepts: “Donning and doffing”, “Training ICU skills”, and “Being instructed in new devices”. Teaching material such as video sequences was provided, and techniques were practiced under supervision.

“I […] also train hygiene concepts. How do I first safely get out of the protective equipment without contaminating myself and second how do I put it off properly and put it back on right without contaminating myself (breathing). That was a huge hygiene training.”—Elisabeth Huber, senior clinician

The clinician showed the effort involved in the training measures by non-verbal expression (breathing). She empathized with her colleagues in daily practice and recapitulated the new established (“huge”) strains of doffing and donning. Clinicians performed as instructors and devised didactic tools to train their staff.

Besides, they had to ensure the care of a potentially large number of critically ill patients and (re)activated all available or newly purchased equipment, especially home care ventilator, injection pumps and hemodialysis machines.

“[We] also thought about using devices in an emergency for things that might not necessarily be used otherwise because they are older or-. Transport monitors, for example, for normal ventilation, if that’s what is needed.”—Karsten Steffen, senior clinician.

The conditions enabled a pragmatic dealing with equipment and led to a change in its valuing. The use of outdated equipment (*“We pulled old ventilators out of the basement”*—Kerstin Jäger, nurse) also triggered fears and hesitation among several participants regarding the upcoming situation. In doing so, nurses in particular expressed doubt to assure the quality of care.

Experiences on building up knowledge and skills were ambivalent: while HCPs considered it valuable, ICU leaders were challenged with organizing care, as well as in their credibility.

### Ambivalence in Infection Prevention and Control

The category “Dealing with personal protective equipment” (PPE) consists of three concepts relating to organizational, operational and individual aspects. Participants reported a lack and rationing of PPE. They had to use respirators and protective gowns more than once, despite previous hygiene regulations. This resulted in negative emotions.

“That’s a no go for me that I should wear a mask the entire shift um yes after so if the mask is wet, it no longer works as it should. I find it just - I don’t know - disgusting hm [(sighs).”—Svenja Fischer, nurse

New routines were experienced as contradictory to established work standards. As a result, they felt constantly confronted with ambivalence.

The shortage of equipment changed thinking and acting.

“In other words, we tried to be much more economical with it [PPE] and to consider it as a valuable resource. That’s a radical change in thinking, because something like that, you didn’t think about it at all before.”—Sebastian Hansen, clinician

Shortages were not generally negative for HCPs; they offered the opportunity to look at the work environment from a different perspective than in pre-pandemic times.

Participants reported that the equipment had to be stored in locked rooms. It was exclusively handed out authorized to each HCP per shift. This also involved the implementation of new procedures and was also experienced as burdensome.

“I considered that to be very stressful. Counting every single face mask every day, handing them out for signatures […].”—Thomas Steiner, head clinician

We observed that with an increase in PPE instructions from hospital managers, HCPs perceived more burden. A shortage of protective equipment highlighted the seriousness of the situation for them.

Under these conditions, new procurement opportunities were identified.

“Then it happened that our janitor service […] (when) the hardware stores were still open, went out and bought masks there, so that we had some in stock.”—Sebastian Hansen, clinician

In addition, HCPs became active themselves. As this clinician reported, HCPs made efforts such as purchasing from hardware stores or using the private neighbor’s 3D printer to provide PPE.

“I can only hope from this whole thing that it remains sustainable, that we in Germany start […] that we ourselves can again provide something in stocks of protective masks, gowns, glasses and gloves. Because the fact that this is becoming so extreme that we don’t have any equipment that has been thrown away for years as disposable material is really frightening.”—Hanna Läufert, nurse, ward manager in absence

Dealing with PPE was a challenge with ambivalent consequences: HCPs experienced the deficiency and management control measures as irritating and frightening. However, the measures also led HCPs to find own solutions.

### Ambivalence in Supporting and Being Supported

A further category covered the process domain of “Perceiving mutual support.” By negotiating ICU structures participants reported perceiving mutual support by family and friends and experienced collegiality through teamwork. They got free drinks and food, massages or even supervision on correct donning and doffing. Some of the supportive services contrasted with challenging tasks that were ahead or expected of the participants in this phase of the pandemic.

However, the members of the teams also supported each other as one nurse reported:

“I really noticed that there were colleagues who were really scared. It really got to their psyche, it was unbelievable. We also had to really take care of a friend of mine. So we consciously talked on the phone with each other every day.”—Tina Hirsch, nurse, ward manager

The HCPs showed themselves to be thoughtful and empathetic. Sharing fears under the conditions bonded the teams and promoted interprofessional cooperation.

Participants mentioned family and friends as an important resource. They listened to each other, were attentive or took care of their children.

“Of course I really have an environment at home where I can actually talk about it.”—Sophie Schünemann, nurse, ward manager

The private sphere was experienced as a place of personal retreat where, ideally, it was possible to distance oneself from everyday work. However, private life was not mentioned in all interviews. For some, colleagues became an important resource. These participants talked about experiencing support from colleagues and about working in a (interprofessional) team. They mentioned changes in teams through new colleagues and structures, but also on moving closer together. In particular, they appreciated teamwork, which was experienced as successful.

“The fact that I had great people on my side. To see, for example, that our heads of intensive care, a woman and a man, were so cool in their work, but also that all my senior clinicians were ready to help, […]. Luckily, it also worked out really well that there was a real team spirit and that you had the feeling that “People, that’s what we studied for, that’s what we were trained for” and that you were able to unite everyone behind this flag.”—Martin Kurtz, head clinician

Participants barely used the psychological support services offered by the hospitals.

“Furthermore, because it played a role in mental health, we used psychological support for the team, which was surprisingly little accepted.”—Elisabeth Huber, senior clinician

The comment of the senior clinician expressed ambivalence between perceived need and utilization of support services.

In turn, hierarchical structures were partially less important in moments of mutual support. However, leaders and persons with more working experience were considered role models who were admired by others. They were able to inspire and motivate within the teams. The participants, at least, mentioned only personalities who they experienced as particularly unconcerned and relaxed in the situation. This led to implicit expectations that could cause further ambivalent tensions in the teamwork.

## Discussion

We examined ICU HCPs’ experiences on professional action at the beginning of the COVID-19 pandemic in Germany. The focus was to reveal implicit principles that structured social practice and interaction of HCPs. Using CGT required an imaginative understanding of the studied phenomenon. This approach *“assumes emergent, multiple realities; indeterminacy; facts and values as linked; truth as provisional; and social life as processual.”*^13^

Five main process categories were identified from the interviews with ICU HCPs: initiating and managing operational changes, managing information, building up knowledge and skills, dealing with personal protective equipment and perceiving mutual support. From the perspective of ICU HCPs, a complex field of ambivalence unfolded between routines of a pre-pandemic normality and pragmatic (restructuring) concepts developed quickly in a new normality of a pandemic-expecting “daily routine of preparation”. Despite experiencing working in “chaos” to prepare for a “disastrous” situation, the involved HCPs retained agency. Dealing with ambivalences is a field of tension between personal and institutional dimensions. It offers possibilities for change and development.

In our data analysis, we identified the negotiation of social practice as a central process under persistent ambivalences. Ambivalence was the recurrent subject within the categories developed.

Ambivalences at the individual (ICU HCPs), sub-institutional (ICU) and institutional (hospital) level ensured that (inter-)actions were constantly renegotiated, leaving ICU HCPs without consistent guidelines for the critical care of COVID-19 patients. Hallgreen et al,^23^ described feelings of ambivalence and uncertainty about working in the ICU, because high demands were placed on nurse anaesthetists’ as professionals to adapt to their employer’s needs. In their interview study, participants described a lack of information from their managers and a short and unstructured introduction to ICU work, which gave rise to feelings of powerlessness.^23^

When pandemic-related measures were mentioned, participants tended to speak of uncertainty and effort, for example, uncertainty about whether their units’ planning and resources would be sufficient to ensure the delivery of high quality patient care throughout the pandemic.^24^ This may be why they used militaristic metaphors and heroic narratives.

Our findings regarding experiences and views from ICU processes at the beginning of the pandemic are consistent with previous published studies.^25-29^ Billings et al^29^ reported in a qualitative meta-synthesis that participants across the included studies were deeply concerned about their own and/or others’ physical safety. This was greatest at the beginning of pandemics and exacerbated by inadequate PPE, insufficient resources, and contradictory information. Frontline HCPs struggled with high a workload and long shifts. The relationships with families, colleagues, organizations, media and the wider public were sometimes strained and could be experienced concomitantly as sources of support.^29^

Authors of a recent rapid review^27^ recommended that coping strategies for HCPs should be assessed and promoted as well as that sufficient PPE should be provided in order to *“mitigate […] negative psychological responses of”* HCPs.^27^ Adjustments of hospital infrastructure to COVID-19 (e.g., sufficient staff, keeping teams and working schedules stable, comprehensive understanding of COVID-19 and continuous provision of proper knowledge) could support HCPs.^28,30^

Our participants used the interview as an opportunity for personal voice. Understanding the negotiation processes gained by ICU HCPs can lead to recommendations for action.

During a time of intense workload for people working in acute care, we succeeded in recruiting 39 HCPs, from different regions in Germany, from general to the university hospital level, right before, during or after the first wave of COVID-19 patients. Both nurses and clinicians were included, reflecting everyday work on ICU.

The sample consisted of mainly white German-speaking persons. We have to mention that persons with a migrant background are increasingly working in the healthcare sector in Germany. Forthcoming research needs to examine their perspectives, too.

Further, our sampling strategy was likely to identify participants who were highly motivated or particularly concerned about the pandemic.

Data collection and analysis was organized in an ongoing circle allowing for maximum openness and for adjusting the design in the course of data collection and data analysis.^13^

As interviews were conducted using telephone or video conferencing systems, it was partially difficult to build a trusting relationship with participants, since non-verbal cues could not be obtained completely.

The subsequent translation of the interview data carries the risk of losing or alienating the original meaning.^31^ We tried to transfer idioms and colloquialisms with English equivalents.

Gender-specific dynamics during data collection cannot be excluded, since only female researchers conducted the interviews.^32^ The varying degree of experience in conducting qualitative research or prior work experience in healthcare (four/seven interviewers) might have influenced the data collection and analysis, too. However, the heterogeneity of the research team facilitated data interpretation adopting different perspectives.

Our study highlighted how ICU staff negotiated social practice and interaction at the beginning of the pandemic in a complex field of ambivalence where processes and interactions were constantly renegotiated. We demonstrated that ambivalence unfolds between routines of pre-pandemic normality and pragmatic restructuring concepts at the beginning of the pandemic. According to Lüscher,^11^ ambivalences are not a priori considered undesirable, disturbing or disadvantageous, rather guide actions. This is an important difference to the understanding in everyday language. Nevertheless, many people perceive ambivalences as burdensome.^11^ We observed these perceptions among our participants. Lüscher^11^ suggests to investigate the reasons for this impression and not to attribute ambivalences “negatively” from the outset. Dealing with ambivalences is a field of tension between personal and institutional dimensions. It offers possibilities for change and development. We showed how experiences of ambivalence guided actions leading to changes in initiating and managing operational structures, managing information, building up knowledge and skills, dealing with PPE and perceiving mutual support. The experience of ambivalence might be inherent to the work of HCPs even in non-pandemic times. Our study showed that it is important that HCPs can deal with ambivalences in a constructive way. Institutions can help HCPs in developing resilience and in initiating change processes and innovations on their own by establishing the prerequisites for transparency, communication and appreciation.

In the meantime, hospitals in Germany have already been confronted with further waves of COVID-19 patients. We do not know whether the experiences of ambivalence as expressed by our study participants changed and/or were perceived as a constructive or destructive factor during the pandemic in retrospective. In our further research, we will adopt a longitudinal perspective to trace changes in experiencing ambivalence by ICU HCPs and its impact on their social practices and interactions.

## Supplemental Material

sj-pdf-1-inq-10.1177_00469580221081059 – Supplemental Material for Intensive Care Units Healthcare Professionals’ Experiences and Negotiations at the Beginning of the COVID-19 Pandemic in Germany: A Grounded Theory Study Click here for additional data file. Supplemental Material, sj-pdf-1-inq-10.1177_00469580221081059 for Intensive Care Units Healthcare Professionals’ Experiences and Negotiations at the Beginning of the COVID-19 Pandemic in Germany: A Grounded Theory Study by Madlen Hörold, Karl Philipp Drewitz, Julia Piel, Ilona Hrudey, Magdalena Rohr, Vreni Brunnthaler, Claudia Hasenpusch, Angela Ulrich, Niklas Otto, Susanne Brandstetter and Christian Apfelbacher in INQUIRY: The Journal of Health Care Organization, Provision, and Financing

sj-pdf-2-inq-10.1177_00469580221081059 – Supplemental Material for Intensive Care Units Healthcare Professionals’ Experiences and Negotiations at the Beginning of the COVID-19 Pandemic in Germany: A Grounded Theory Study Click here for additional data file. Supplemental Material, sj-pdf-2-inq-10.1177_00469580221081059 for Intensive Care Units Healthcare Professionals’ Experiences and Negotiations at the Beginning of the COVID-19 Pandemic in Germany: A Grounded Theory Study by Madlen Hörold, Karl Philipp Drewitz, Julia Piel, Ilona Hrudey, Magdalena Rohr, Vreni Brunnthaler, Claudia Hasenpusch, Angela Ulrich, Niklas Otto, Susanne Brandstetter and Christian Apfelbacher in INQUIRY: The Journal of Health Care Organization, Provision, and Financing

## Appendix

INQ-21-0484 Pandemic negotiation of German ICU healthcare professionals
