# Supplementary material for: Intensive Care Units Healthcare Professionals’ Experiences and Negotiations at the Beginning of the COVID-19 Pandemic in Germany: A Grounded Theory Study
Source: Inquiry. 2022 May 6;59:00469580221081059. doi: 10.1177/00469580221081059 (PMC9082755; doi:10.1177/00469580221081059)
Supplement: sj-pdf-1-inq-10.1177_00469580221081059 – Supplemental Material for Intensive Care Units Healthcare Professionals’ Experiences and Negotiations at the Beginning of the COVID-19 Pandemic in Germany: A Grounded Theory Study [file sj-pdf-1-inq-10.1177_00469580221081059.pdf]

Interview protocol

General data

Date:	Duration:
Follow up:	☐ has already been agreed<br>☐ contact required again

Sociodemographic data

Sex:	☐ female<br>☐ male<br>☐ divers
Age:	
Work location/region (maximum federal state, otherwise only whether metropolis/large city/rural region):	
Type of hospital (university hospital, maximum care hospital, centralized care hospital, basic and regular care hospital)	
Professional background and duration of work experience (education, training, ...):	

Further information

Interview atmosphere:
Interview interaction:
Keywords on personal relationship:
Other:
