# Supplementary material for: Intensive Care Units Healthcare Professionals’ Experiences and Negotiations at the Beginning of the COVID-19 Pandemic in Germany: A Grounded Theory Study
Source: Inquiry. 2022 May 6;59:00469580221081059. doi: 10.1177/00469580221081059 (PMC9082755; doi:10.1177/00469580221081059)
Supplement: sj-pdf-2-inq-10.1177_00469580221081059 – Supplemental Material for Intensive Care Units Healthcare Professionals’ Experiences and Negotiations at the Beginning of the COVID-19 Pandemic in Germany: A Grounded Theory Study [file sj-pdf-2-inq-10.1177_00469580221081059.pdf]

Interview guide

Initial question

Today, I would like to talk to you about the phase of preparations for the care of COVID-19-infected ICU patients in your everyday work. I am interested in your personal perspective about the current situation. Therefore, I would simply like to ask you to tell me how you experience the phase of preparation for the care of COVID-19 infected patients. In doing so, you can initially take as much time as you like. I will only interrupt you if something is unclear or incomprehensible. Otherwise, you can talk about whatever comes to your mind.

Thematic structuring questions for the first interview (preparation phase)

How do you currently prepare in the hospital for the expected high number of persons requiring monitoring or intensive care with COVID-19?

Prompts

- Infection control measures?
- "Disaster plans" (triage, etc.), counselling?
- Assessment of capacity-limiting factors: staff availability; critical equipment; bed spaces; management and coordination system
- Sufficient preparation through education, training, and work experience?
- Information from leadership, policy makers, the professional community?

How do you collaborate with colleagues and members of other professional groups?

Prompts

- Evaluation of the team on the ward?
- Changes in the team through preparation?
- Participation in the training of new employees?
- Evaluation of the cooperation in the clinic in general?

In preparing for this situation, to what extent do you experience fears or conflicts? Decision conflicts?

Prompts

- Where do you see decision conflicts in the current situation?
- What do you use to guide your decisions about which patients to treat with intensive care in the first place?
- What do you do when you have to decide which patient should be ventilated and who should not?
- Who should be involved in such a decision?
- How unambiguous do you consider the decision-making process to be?
- Do you already know this from your own experience (or from colleagues)?

To what extent are you currently exposed to other specific burdens?

Prompts

- Psychological stress?
- Influence of stress on activity?
- Evaluation of the help offered by the hospital
- What gives you strength/stability in the current situation?
- Coping strategies?
- Personal contacts
- What ideas do you (already) have for dealing with these?

General prompts

- Can you give me an example?

- Please describe what you think this means
- What specifically do you think is the reason for this?
- How does it make you feel?
- What does it do to you?
